# Impact of the Ceiling-Mounted Radiation Shielding Position on the Physician's Dose from Scatter Radiation during Interventional Procedures

**DOI:** 10.1155/2018/4287973

**Published:** 2018-01-30

**Authors:** Lucie Sukupova, Ondrej Hlavacek, Daniel Vedlich

**Affiliations:** ^1^Department of the Director, Institute for Clinical and Experimental Medicine, Videnska 1958/9, 140 21 Prague 4, Czech Republic; ^2^Radiodiagnostic and Interventional Radiology Department, Institute for Clinical and Experimental Medicine, Videnska 1958/9, 140 21 Prague 4, Czech Republic

## Abstract

The effect of the ceiling-mounted radiation shielding on the amount of the scatter radiation was assessed under conditions simulating obese patients for clinically relevant exposure parameters. Measurements were performed in different projections and with different positions of the ceiling-mounted shielding: without shielding; shielding closest to the patient; and shielding closest to the physician performing the procedure. The protection provided by the shielding was assessed for cardiology when the femoral access is used and for radiology when the physician performs the procedure in the abdominal area. The results show that the use of the ceiling-mounted shielding can decrease the dose from the scatter radiation by 95% at the position of the performing physician. In cardiology, the impact is more pronounced when the left oblique projection is used. In radiology, a large decrease was observed for right oblique projections, compared to cardiology. The ceiling-mounted shielding should be placed as close to the physician as possible. The idea of creating the largest radiation shadow by placing the radiation shielding as close to the patient as possible does not provide as effective radiation protection of the operator as it might be thought.

## 1. Introduction

It is widely accepted that low dose exposures can increase cancer risk, and as a consequence the scatter radiation produced by patients during fluoroscopy guided procedures may present a risk for performing physicians [1]. Physicians performing interventional procedures in close proximity to patients are exposed to potentially low levels of scattered dose for long time period [2], making radiation protection of the interventional staff an important issue [3, 4]. There are practical ways of reducing occupational doses to interventional staff, including the use of protective shielding. Protective shielding may be personal—aprons, thyroid shields, and glasses—or could be part of the X-ray system and its accessories as ceiling-mounted shielding, table shielding with vertical extension, disposable pads, or shielding placed over patients [5–10]. Doses to both staff and patients can also be reduced through the use of modified angiography system setups (frame rate, X-ray field size, spectral filtration, projection, etc.). These are described in [5] and will not be discussed here.

In some situations, shielding placed correctly can significantly improve the radiation protection of the staff; Fetterly et al. [11] reviewed effects of different types shielding and its influence on protection from scatter radiation. Fetterly et al. were dealing with the positioning of the lower, middle, and upper body shielding and its influence on protection from scatter radiation. The authors measured doses by simulating procedures performed on angiography systems but only in the posteroanterior (PA) projection, which is not commonly used in cardiology, and sometimes radiology procedures. Physicians use projections including left anterior oblique (LAO) and right anterior oblique (RAO), often tilted in the cranial (CR) or caudal (CD) directions. In the LAO projection, the X–ray beam is entering the patient from the right hand side, and in the CR projection the X-ray beam is entering from the lower part of the patient's body towards the head direction.

In this study, the influence of the ceiling-mounted shielding and its positioning on the occupational doses of performing physicians due to the scatter radiation from different projections under conditions simulating the exposures during interventional procedures was investigated.

## 2. Materials and Methods

Two angiography systems, each equipped with a ceiling-mounted radiation shielding, were chosen for this study. The first, Artis Q (installed in 2016, Siemens, Erlangen, Germany) has a 25 cm flat panel detector and is used for cardiac procedures. The cine mode Coro_CARE (dose saving mode) was chosen for measurement, each cine acquisition running for 4 s with the frame rate 15 fr/s, as used clinically. The exposure parameters were set automatically by the use of the automatic dose rate control (ADRC), which is used on both angiography systems. The exposure parameters were as follows: 86–96 kVp, 340–780 mA, pulse width 5.2–9.0 ms, 0.0 mm Cu additional filtration, and small and large focus.

The second system, Artis Zee (installed in 2012, Siemens, Erlangen, Germany), with a flat panel detector of 48 cm diagonally is used for interventional radiology. On Artis Zee, the mode for abdominal procedures (biliary tract dilation, nephrostomy) was chosen for measurement, with each cine acquisition running for 4 s with the frame rate 7.5 fr/s (this is intentionally higher for better measurement accuracy: 1 single frame or 1 fr/s is used routinely). The exposure parameters chosen by the ADRC were as follows: 81–115 kVp, 478–668 mA, pulse width 36–39 ms, 0.0 mm Cu additional filtration, and large focus.

The attenuation of the patient was simulated by the anthropomorphic Alderson male adult RANDO phantom, the torso with head, but without arms and legs. Due to the relatively small size of the RANDO phantom (much smaller than our average patient of 85 kg), higher attenuation was simulated by adding the PMMA slabs to the RANDO phantom. For the cardiology system, 4 cm of the PMMA was placed above the phantom and 2 cm under the phantom. The RANDO phantom with added PMMA slabs simulates a patient weighting between 85 and 90 kg, which corresponds more closely to our patient group. For the radiology system, an additional 2 cm of the PMMA was placed both right and left side of the RANDO phantom to simulate larger patients. Larger patients produce more scatter radiation, because they require more radiation to be used (a patient with the PA diameter of 29 cm requires about 200% more radiation than a patient with the diameter of 24 cm [12]) and hence produce much more scatter [12].

**Figure 1:**
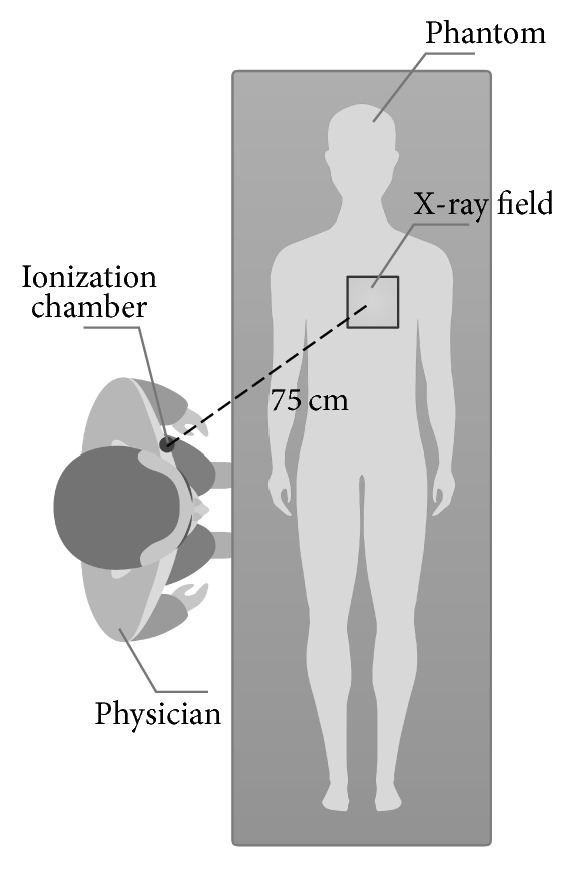
The geometry of measurement on the angiography system for cardiology.

**Figure 2:**
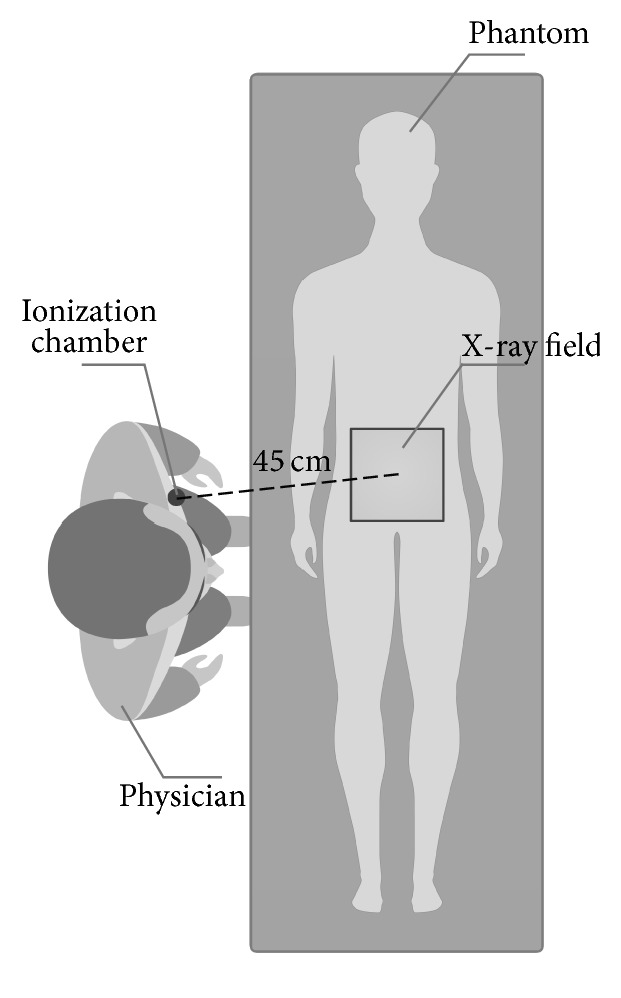
The geometry of measurement on the angiography system for radiology.

The amount of scatter radiation was measured by the Radcal 9095 system (Radcal Corporation, Monrovia, California, USA) with the cylindrical 1800 cc ionization chamber 10X6-1800 (the active volume 1 800 cm^3^). Correction for energy dependence [13] and angle dependence [14] is negligible. The measurements were performed in the dose accumulation mode. The geometry was as follows: the patient table with the RANDO phantom placed 90 cm above the floor, source to table distance of 60 cm, and source to detector distance of 110 cm. The centre of the ionization chamber was placed 135 cm above the floor which corresponds to the chest of the performing physician. The distance of the centre of the ionization chamber from the X-ray field was different for cardiology and radiology and is shown in Figures 1 and 2.

The radiation shielding tested was the ceiling-mounted shielding for the upper body with equivalent of 0.5 mm Pb (OT54001, Mavig, Germany) together with a panel curtain 0.5 mm Pb equivalent (OT94001, Mavig, Germany) which provides a patient contour cutout. At all the measurements, a table-mounted (undertable) shield for the lower body with equivalent of 0.5 mm Pb was also used, but only in one position.

For the cardiology system, the heart of the RANDO phantom was irradiated. The ionization chamber was placed 75 cm from the centre of the X-ray field, which corresponds to the chest of the physician when femoral access is used (illustrated in Figure 1). The X-ray field size at 60 cm from the X-ray tube (at the interventional reference point distance) was 10 cm × 10 cm.

For the radiology system, a lower abdominal area of the RANDO phantom was irradiated. The ionization chamber was placed 45 cm from the centre of the X-ray field, which corresponds to the chest of the physician (illustrated in Figure 2). The X-ray field size at 60 cm from the X-ray tube (at the interventional reference point distance) was 18 cm × 18 cm.

The amount of the scatter radiation was measured for the position of the physician standing at the right hand side of the patient at the physician's left chest side for three positions of the ceiling-mounted shielding:

- Without the shielding
- With the shielding placed closest to the patient (the dose measured relatively far behind the shielding)
- With the shielding placed closest to the physician (the dose measured just behind the shielding).

**Figure 3:**
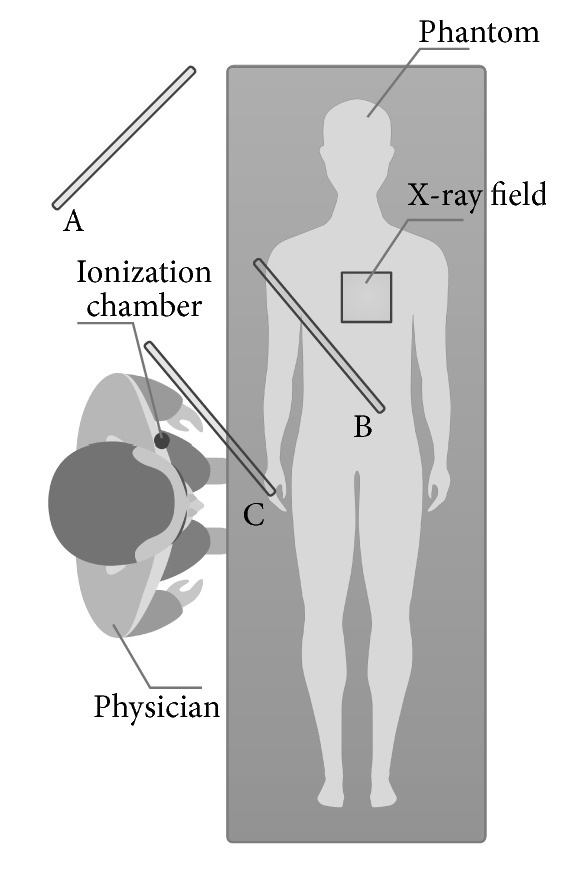
Different positions of the ceiling-mounted shielding for cardiology.

**Figure 4:**
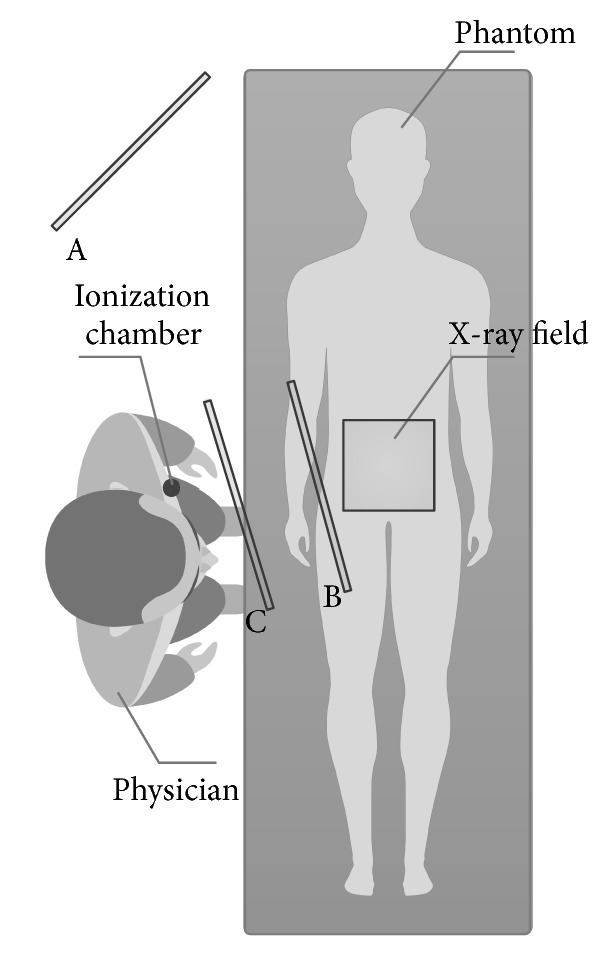
Different positions of the ceiling-mounted shielding for radiology.

All the three positions are illustrated in Figure 3 for cardiology and in Figure 4 for radiology. Unfortunately, in some projections, for example, steep RAO projections, the measurements in positions B and C were the same, because the position of the ionization chamber and the radiation shielding was complicated due to the presence of the flat panel detector.

## 3. Results and Discussion

### 3.1. Results

**Figure 5:**
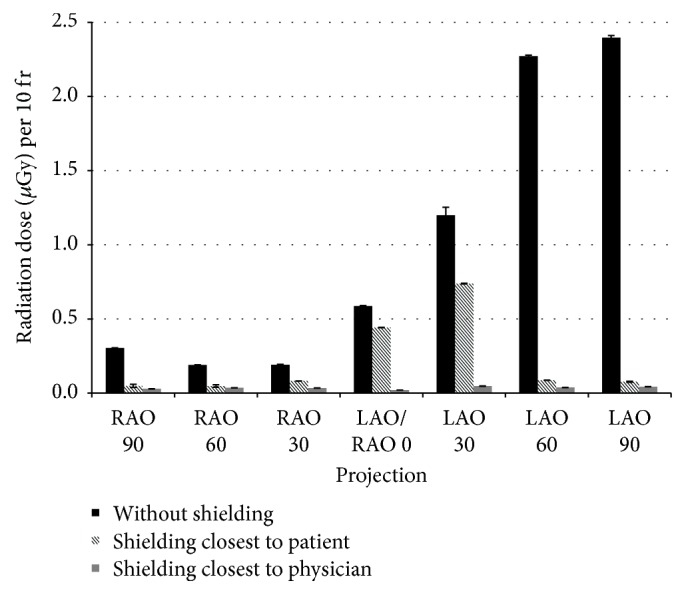
Doses from the scatter radiation in cardiology for RAO 90° to LAO 90°, CD/CR 0°.

**Figure 6:**
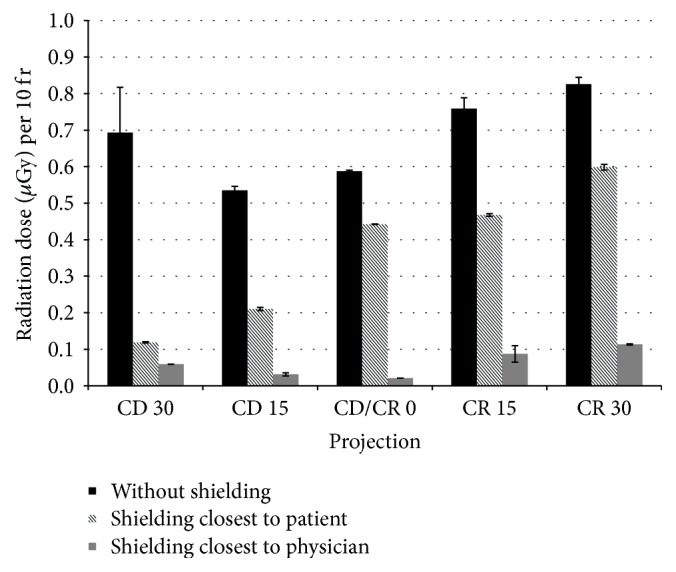
Doses from the scatter radiation in cardiology for LAO/RAO 0°, CD 30° to CR 30°.

For cardiology, the doses from the scatter radiation per 10-frame acquisition were measured for 22 different projections that are routinely used in cardiology. Each measurement was performed twice. The results are given in Table 1. Digits in bold do not differ significantly at 0.05 level of significance. For some projections, the doses with their standard deviations are shown in Figures 5 and 6.

For radiology, the doses from the scatter radiation were measured for 9 different projections that are routinely used. Doses are included in Table 2. All doses were taken for a 10-frame acquisition. For some projections, the values are the same for shielding placed close to the patient as for shielding placed close to the physician due to the complicated geometry (obstruction caused by the position of the flat panel detector or X–ray tube). In these cases, there are only two values in the cell.

The dose to the phantom for each projection was driven by the ADRC. It is known that the dose for steep projections is higher [15–17]. Therefore, the doses from the scatter radiation for each projection were normalized to the *P*_KA_ values that were gained during the measurement from the KAP-meter (with uncertainty 5%) and are shown in Table 3 for cardiology.

In the same way, as for cardiology, the doses from the scatter radiation for each projection were normalized to the *P*_KA_ values and are shown in Table 4.

### 3.2. Discussion

In cardiology, the doses to the physician from the scatter radiation are the highest from LAO projections. In these projections the X-ray tube is on the right side of the patient, therefore close to the physician when the right side radial or femoral access is used. The dose from the scatter radiation can go up to 2.8 *μ*Gy per 10 fr when the ceiling-mounted shielding is not used (Table 1). However, the dose from scatter radiation appears to be lowered by more than 95% when the ceiling-mounted shielding is used. If the shielding is placed as close to the physician as possible (as opposed to placing the shielding as close to the patient as possible), there is another decrease of the dose by 50% in LAO projections. This decrease with the positioning of the shielding was not observed for RAO projections. With regards to LAO/RAO 0°, CD/CR projections, the use of the shielding can lower the dose by 30–80% when placed as close to the patient as possible and by another 20–80% when placed as close to the physician as possible.

Doses from scatter radiation for the position of the shielding closest to the patient and closest to the physician were not significantly different at the level 0.05 for some projections (these values are in bold in Table 1), which may be caused by the fact that only two measurements in each position were performed.

The highest dose from all projections was measured for LAO 30°, CR 30° projection, when the X-ray tube is close to the physician. In this projection, the dose to the physician can by lowered by 94% when the shielding is placed as close to the physician as possible.

In radiology, the doses to the physician from the scatter radiation are also the highest from LAO projections, when the X-ray tube is on the right side of the patient. In LAO projections, the dose can be as high as almost 140 *μ*Gy per 10 fr. The dose could be even higher, for example, for LAO 90°, but the value could not be measured due to the high dose rate. The dose from the scatter radiation can be lowered by 98% when the ceiling-mounted shielding is used. This large decrease was observed for RAO projections too, whereas, in cardiology, the large decreases were seen for LAO projections only. If the shielding is placed as close to the physician as possible compared to placing the shielding as close to the patient as possible, there is another decrease of the dose by 85% in LAO projections. For some projections (LAO 30°, CD 20°; LAO 90°, CR/CD 0°), the dose from the scatter radiation without the use of the shielding could not be measured due to the high dose rate.

The dependence of the dose on the shielding positioning is smaller for some steeper projections in radiology, for example, LAO 30°, CD/CR 20°, because the freedom in the positioning is limited by the small space between the X-ray tube and the physician or the detector and the physician.

The doses from scatter radiation are approximately 35–180 times higher for radiology than for cardiology, depending on the projection. The physician-interventional radiologist usually stands much closer to the exposed area of the patient (the abdominal area in our case), so his dose from scatter radiation is much higher. As the doses from scatter radiation are much higher for radiologists, the eye lens of radiologists may be at higher risk of cataracts making the wearing of glasses with Pb equivalent particularly important for radiologists.

The use of the ceiling-mounted shielding is crucial at LAO projections when the performing physician is standing at the right hand side of the patient. The amount of the scatter radiation for the physician in LAO projections is more than 10 times higher than in the RAO projections.

The physician's dose depends on the *P*_KA_ value used in the projection, as mentioned above, so the dose from scatter radiation in the steeper projection is higher. The doses from scatter radiation normalized to *P*_KA_ were determined and are included in Tables 3 and 4.

The values in Tables 3 and 4 show that doses from scatter radiation normalized to *P*_KA_ are higher for radiology than for cardiology. There may be a number of reasons why. In the case of abdomen imaging, which was simulated here, the physician stands closer to the source of scatter radiation. Other causes might be the larger X-ray fields that are used (10 cm for cardiology versus 18 cm for radiology) and also as more attenuating areas are investigated (the heart in lungs versus the abdominal area), more scatter radiation may be produced.

The results were compared with results from the study of Kuon et al. [17] on invasive cardiology. There was good agreement between their and our results. The physician receives the lowest dose from scatter radiation in RAO 20–30° projections. On the other hand, he receives the highest dose from LAO 60–70°, CD 0–20° projections. The normalized values of scatter dose to *P*_KA_ were of the same order for their and our study, for example, LAO/RAO 0°, CR/CD 0° 0.023 versus 0.022 *μ*Gy/*μ*Gy *∗* m^2^, RAO 90°, CD/CR 0° 0.017 versus 0.011 *μ*Gy/*μ*Gy *∗* m^2^, LAO 90°, CR/CD 0° 0.062 versus 0.086 *μ*Gy/*μ*Gy *∗* m^2^, and LAO 60°, CD 10–20° 0.072 versus 0.081 *μ*Gy/*μ*Gy *∗* m^2^.

The results correspond with results from the study of Fetterly et al. [11], where the authors showed that in the LAO/RAO 0°, CR/CD 0° projection the upper body shielding placed closer to the femoral access, and therefore to the operator, provides better radiation protection than the shielding placed 20 cm farther from the operator and closer to the patient.

If the ceiling-mounted shielding is placed closer to the patient, a larger solid angle is shielded but with lower efficiency. On the other hand, if the shielding is placed close to the operator, a smaller solid angle is shielded but with higher efficiency. This should be taken into account when more people are present in the catheterization laboratory.

This study has its limitations. The measurements were performed for one main operator with changed positions of the upper body shielding, but one position of the lower body shielding. The situation would be different when there are two or more operators or other staff in the catheterization laboratory.

Another limitation is that the operator's position may be different for procedures performed from different access, for example, the patient's left side in cardiology, typical in electrophysiology, when pacemakers are implanted. Similarly in radiology, different approaches will be used when the procedure is performed from the jugular vein access or for procedures outside of the abdominal area, for example, limbs.

The measurements were performed for one size of the phantom and one size of the X-ray field. The results would be different for patients of different sizes and also for different sizes of X-ray field. For smaller patients and smaller fields, less scatter would be expected.

## 4. Conclusion

The use of radiation shielding has a significant impact on the amount of scatter radiation at the position of the performing physician, so the use of radiation shielding should be recommended in all projections.

The dose of the physician performing interventional procedures may be reduced significantly, when the ceiling-mounted shielding is used correctly during the procedures. The influence of the position of the ceiling-mounted radiation shielding on the physician's dose is more pronounced for LAO projections and also for LAO projections tilted in the CD/CR direction. For cardiology, the position of the ceiling-mounted shielding in RAO projections does not affect the dose from the scatter radiation as significantly as it does for LAO projections. However, this is not the case for radiology, where the position of the shielding affects the dose from the scatter radiation in all projections performed on the abdominal area.

In summary, the ceiling-mounted shielding should be used wherever possible, placed as close to the physician as possible. This approach should be applied only to situations, when there is only one operator in the catheterization laboratory where upper and lower body shielding is used. The situation would be different, when there are more people in a catheterization room. The idea of creating the largest radiation shadow by placing the radiation shielding as close to the patient as possible might be used in these situations but we were not dealing with this aspect in our study.

## Figures and Tables

**Table 1:** Doses (*μ*Gy) from scatter radiation per 10 frames in cardiology for different projections (in each cell, dose without the use of shielding, with shielding placed close to the patient, and with shielding placed close to the physician. Digits in bold do not differ significantly at 0.05.).

	RAO			0°	LAO		
	90°	60°	30°		30°	60°	90°
CD							
30°			0.77/0.17/0.08	0.69/0.12/0.06	2.76/0.28/0.12		
15°		0.18/**0.07/0.03**	0.27/0.10/0.04	0.54/0.21/0.03	1.73/0.73/0.06	2.36/**0.10/0.05**	
0°	0.30/**0.05/0.03**	0.19/**0.05/0.04**	0.19/0.08/0.03	0.59/0.44/0.02	1.20/0.74/0.05	2.27/0.09/0.04	2.40/**0.08/0.04**
CR							
15°		0.22/**0.06/0.03**	0.45/0.28/0.06	0.76/0.47/0.09	2.59/0.46/0.07		
30°			0.74/0.38/0.13	0.83/0.60/0.11	4.51/0.97/0.29		

**Table 2:** Doses (*μ*Gy) from scatter radiation per 10 fr in radiology for different projections (in each cell, dose without the use of shielding, with shielding placed close to the patient, and with shielding placed close to the physician. Hyphens mean that the values could not be measured because the dose rate was too high. When there are only two values in the cell, the measurements with shielding placed close to the patient and with shielding placed close to the physician were the same. All the values differ significantly at 0.05.).

	RAO		0°	LAO	
	90°	30°		30°	90°
CD					
20°		28.38/1.43		-* *-* *-/4.45/3.55	
0°	10.72/2.67	34.68/1.55	48.35/4.64/0.89	137.31/15.97/2.32	-* *-* *-/5.02
CR					
20°		44.35/2.92/2.01		119.69/6.55/4.22	

**Table 3:** Dose from scatter radiation normalized to *P*_KA_ (*μ*Gy/*μ*Gy *∗* m^2^) for cardiology (in each cell dose without the use of shielding, with shielding placed close to the patient and with shielding placed close to the physician).

	RAO			0°	LAO		
	90°	60°	30°		30°	60°	90°
CD							
30°			0.010/0.002/0.001	0.016/0.003/0.001	0.037/0.004/0.002		
15°		0.005/0.002/0.001	0.007/0.003/0.001	0.019/0.007/0.001	0.040/0.017/0.001	0.081/0.003/0.003	
0°	0.011/0.002/0.001	0.006/0.001/0.001	0.007/0.003/0.001	0.022/0.016/0.001	0.037/0.023/0.001	0.086/0.003/0.001	0.086/0.003/0.002
CR							
15°		0.007/0.002/0.001	0.009/0.006/0.001	0.026/0.016/0.003	0.052/0.009/0.001		
30°			0.007/0.003/0.001	0.016/0.012/0.002	0.043/0.009/0.003		

**Table 4:** Dose from scatter radiation normalized to *P*_KA_ (*μ*Gy/*μ*Gy *∗* m^2^) for radiology (in each cell dose without the use of shielding, with shielding placed close to the patient, and with shielding placed close to the physician. When there are only two values in the cell, the measurements with shielding placed close to the patient and with shielding placed close to the physician were the same).

	RAO		0°	LAO	
	90°	30°		30°	90°
CD					
20°		0.021/0.001		-* *-* *-* *-/0.003/0.003	
0°	0.008/0.002	0.026/0.001	0.044/0.004/0.001	0.107/0.012/0.002	-* *-* *-* *-/0.004
CR					
20°		0.034/0.002/0.002		0.090/0.005/0.003	
